# In Silico Design of Peptide Inhibitors Targeting HER2 for Lung Cancer Therapy

**DOI:** 10.3390/cancers16233979

**Published:** 2024-11-27

**Authors:** Heba Ahmed Alkhatabi, Hisham N. Alatyb

**Affiliations:** 1 Faculty of Applied Medical Science, King Abdulaziz University, Jeddah 22254, Saudi Arabia; 2 Hematology Research Unit (HRU), King Fahd Medical Research Center (KFMRC), Jeddah 22252, Saudi Arabia; 3 Center of Artificial Intelligence in Precision Medicines, King Abdulaziz University, Jeddah 22254, Saudi Arabia; hishamaltayb@gmail.com; 4 Department of Biochemistry, Faculty of Science, King Abdulaziz University, Jeddah 22254, Saudi Arabia

**Keywords:** human epidermal growth factor receptor 2 (HER2), density functional theory (DFT), quantum mechanics/molecular mechanics (QM/MM) simulation, molecular dynamics simulation (MD), HER2-overexpressing cancers

## Abstract

Background/Objectives: Human epidermal growth factor receptor 2 (HER2) is overexpressed in several malignancies, such as breast, gastric, ovarian, and lung cancers, where it promotes aggressive tumor proliferation and unfavorable prognosis. Targeting HER2 has thus emerged as a crucial therapeutic strategy, particularly for HER2-positive malignancies. The present study focusses on the design and optimization of peptide inhibitors targeting HER2, utilizing machine learning to identify and enhance peptide candidates with elevated binding affinities. The aim is to provide novel therapeutic options for malignancies linked to HER2 overexpression. Methods: This study started with the extraction and structural examination of the HER2 protein, succeeded by designing the peptide sequences derived from essential interaction residues. A machine learning technique (XGBRegressor model) was employed to predict binding affinities, identifying the top 20 peptide possibilities. The candidates underwent further screening via the FreeSASA methodology and binding free energy calculations, resulting in the selection of four primary candidates (pep-17, pep-7, pep-2, and pep-15). Density functional theory (DFT) calculations were utilized to evaluate molecular and reactivity characteristics, while molecular dynamics simulations were performed to investigate inhibitory mechanisms and selectivity effects. Advanced computational methods, such as QM/MM simulations, offered more understanding of peptide–protein interactions. Results: Among the four principal peptides, pep-7 exhibited the most elevated DFT values (−3386.93 kcal/mol) and the maximum dipole moment (10,761.58 Debye), whereas pep-17 had the lowest DFT value (−5788.49 kcal/mol) and the minimal dipole moment (2654.25 Debye). Molecular dynamics simulations indicated that pep-7 had a steady binding free energy of −12.88 kcal/mol and consistently bound inside the HER2 pocket during a 300 ns simulation. The QM/MM simulations showed that the overall total energy of the system, which combines both QM and MM contributions, remained around −79,000 ± 400 kcal/mol, suggesting that the entire protein–peptide complex was in a stable state, with pep-7 maintaining a strong, well-integrated binding. Conclusions: Pep-7 emerged as the most promising therapeutic peptide, displaying strong binding stability, favorable binding free energy, and molecular stability in HER2-overexpressing cancer models. These findings suggest pep-7 as a viable therapeutic candidate for HER2-positive cancers, offering a potential novel treatment strategy against HER2-driven malignancies.

## Simple Summary

Human epidermal growth factor receptor 2, a protein found on cell surfaces, is often overproduced in aggressive forms of cancers like breast, gastric, ovarian, and lung cancers. This overproduction can lead to rapid tumor growth and poor patient outcomes. Current treatments target this protein to slow cancer progression, but more effective and precise options are needed. This study explores a new approach by designing small protein-like molecules called peptides to specifically block this receptor. Using advanced computer-based methods, researchers identified and optimized several peptides, selecting the ones most likely to bind effectively to this protein. Through further testing, one peptide showed promise as a potential therapy due to its stable binding and favorable properties. This research could lead to new, targeted treatment options for patients with these types of cancers and contribute to ongoing efforts to improve cancer therapies through precision medicine.

## 1. Introduction

Globally, cancer has become a significant public health concern. According to World Health Organization (WHO) estimations from 2019, cancer ranks either first or second among the major causes of death in 112 out of 183 nations [1]. Cancer remains a leading cause of death globally, with an estimated 1.8 million deaths (18.7%) attributed to it annually. Lung cancer is the most common cause of cancer-related mortality, followed by colorectal (9.3%), liver (7.8%), female breast (6.9%), and stomach (6.8%) cancers [2,3]. Lung cancer, specifically non-small cell lung cancer (NSCLC), accounts for approximately 85% of all cases [4]. The overexpression of HER2 (human epidermal growth factor receptor 2) is a critical determinant in the aggressive advancement of multiple malignancies, including breast, gastric, lung, and colorectal cancers. HER2-overexpressing tumors are linked to unfavorable prognosis and are significant targets for therapeutic intervention. Targeting HER2, due to its involvement in tumor proliferation across several cancer types, presents a viable strategy for treating a wide range of HER2-positive malignancies.

HER-family protein is responsible for abnormal cell growth in breast cancer [5]. The family of receptors known as HER is involved in cell signaling. In the HER family, HER2 plays a crucial function by interacting with other receptors to intensify intracellular signals. It includes several members, such as HER1 (EGFR), HER2 (ErbB2), HER3 (ErbB3), and HER4 (ErbB4), to regulate cell proliferation and differentiation [6]. HER2 is a member of the epidermal growth factor receptor (EGFR) or human ErbB protein family, having tyrosine–kinase activity [7]. Every member of the ErbB family of receptors has a cytoplasmic tyrosine–kinase region, and all but HER2 binds to certain ligands via the extracellular domain [8]. HER2 has the ability to stimulate the development of cancer cells. The overexpression of the HER2 receptor can cause tumors to develop aggressively by transforming normal cells into cancerous cells. It is frequently overexpressed in particular forms of cancer, which also makes it a target for specific treatments [9,10,11,12].

HER2 overexpression is well-established in breast cancer but has also been recognized in other cancers, including lung, gastric, and colorectal cancers. Recent research into HER2-targeted therapies, such as trastuzumab, has extended beyond breast cancer, showing potential benefits in other solid tumors where HER2 alterations contribute to aggressive tumor behavior and poor prognosis [13]. The role of HER2 in breast cancer has been extensively studied, but research on its significance in lung cancer and gastric cancer is still ongoing [14]. HER2-targeted therapies are being explored for patients with HER2 mutations in a variety of tumors, showing potential benefits in treatment [15]. The development of the humanized anti-HER2 antibody trastuzumab spurred more study into HER2-specific antibodies, kinase inhibitors, and dimerization inhibitors for the treatment of cancer [16]. By selectively binding to the HER2 receptor, trastuzumab inhibits the receptor’s activation and signaling cascades. This inhibition increases the efficacy of chemotherapy drugs like doxorubicin and paclitaxel by preventing the growth of cancer cells that overexpress HER2 [17]. While trastuzumab has demonstrated success in breast cancer, its effectiveness in other HER2-overexpressing cancers, such as lung and gastric cancers, remains under investigation [18].

While anti-HER2 inhibitors like trastuzumab have been effective in treating HER2-positive cancers, there is increasing interest in developing smaller, more flexible therapeutic agents, such as peptides. These agents may offer a similar effectiveness with the potential for reduced immune system reactions (immunogenicity) and better tissue penetration. Peptides, due to their smaller size, are often expected to penetrate tissues more effectively than large antibodies like trastuzumab. This study, therefore, presents a thorough approach to designing and optimizing peptide inhibitors specifically targeting HER2. It begins with identifying the target protein and mapping its interactions, followed by designing and mutating peptides to enhance their binding. The peptides are modeled in 3D and docked with the target protein, and their solvent accessibility and binding energies are calculated. Advanced methods including DFT and molecular dynamics simulation are employed to assess the electronic properties and the stability of the peptide–protein complexes over time.

## 2. Literature Review

HER2 (human epidermal growth factor receptor 2) is a transmembrane protein involved in cell signaling pathways that regulate proliferation and differentiation. The overexpression or amplification of HER2 is a characteristic feature of highly aggressive malignancies, including those of the lung, breast, gastric, and ovarian tissues. This change is associated with a lower prognosis and often results in limited therapeutic options for affected patients [19]. Conventional therapies targeting HER2, such as trastuzumab, have demonstrated substantial efficacy in HER2-positive breast cancer. However, their effectiveness is decreased in other malignancies, including lung and gastric cancers [19,20]. These limitations increase the necessity of developing a novel inhibitor against HER2.

Recent advancements in computational biology have simplified the design and evaluation of peptide-based inhibitors. Techniques such as molecular docking, machine learning (ML), molecular dynamics (MD), and hybrid quantum mechanics/molecular mechanics (QM/MM) simulations have been involved in identifying peptide candidates with improved stability and binding affinity [21,22]. These approaches have successfully identified peptides with high binding affinities for HER2, using computational techniques. Machine learning, specifically regression models, has proven effective in predicting peptides’ binding affinities and identifying top candidates for further optimization [23]. Molecular dynamics simulations offer valuable insights into the stability and interaction dynamics of peptide–protein complexes [24]. Additionally, techniques such as FreeSASA and density functional theory (DFT) analyses are crucial for evaluating solvent accessibility, electronic properties, and the stability of peptides. Studies on HER2 focuses on incorporating QM/MM simulations to examine electronic and mechanical interactions at the molecular level, an essential approach for developing potent peptide-based therapeutics [25,26].

## 3. Results

### 3.1. Protein Structure

The structure of receptor tyrosine–protein kinase erbB-2 (HER2) was searched in UniProt, and the reviewed entry with the UniProt ID P04626 was selected. Following this, the crystal structure with the highest resolution, which was also the most recent, was chosen and identified with PDB ID 8JYR. The PDB structure with PDB ID 8JYR, which presents the crystal structure of the anti-HER2 antibody H2Mab-119 in a complex with the HER2 domain I, offers valuable insights into the specific interactions between HER2 and its inhibitor. This structure highlights a distinct motif within the HER2 domain I that is crucial for high-affinity binding by H2Mab-119. The anti-HER2 monoclonal antibody (mAb) H2Mab-119 is a member of the IgG1 kappa class. H2Mab-119 interacts with both normal and cancerous epithelial cells and might have a negative therapeutic impact [27]. Structural analysis of the PDB was performed using visualization by PyMOL version 3.1 to explore the interaction sites between HER2 and the antibody.

It was identified that the PDB file for this structure used non-standard residue numbering for certain residues. For example, in the heavy chain, Lys53A, Asn82A, Ser82B, Leu82B, Tyr100A, Ala100B, and Ala100C were present, and in the light chain, Ser30A, Tyr30B, Gly30C, and Asn30D were found using PyMOL.

**Figure 1:**
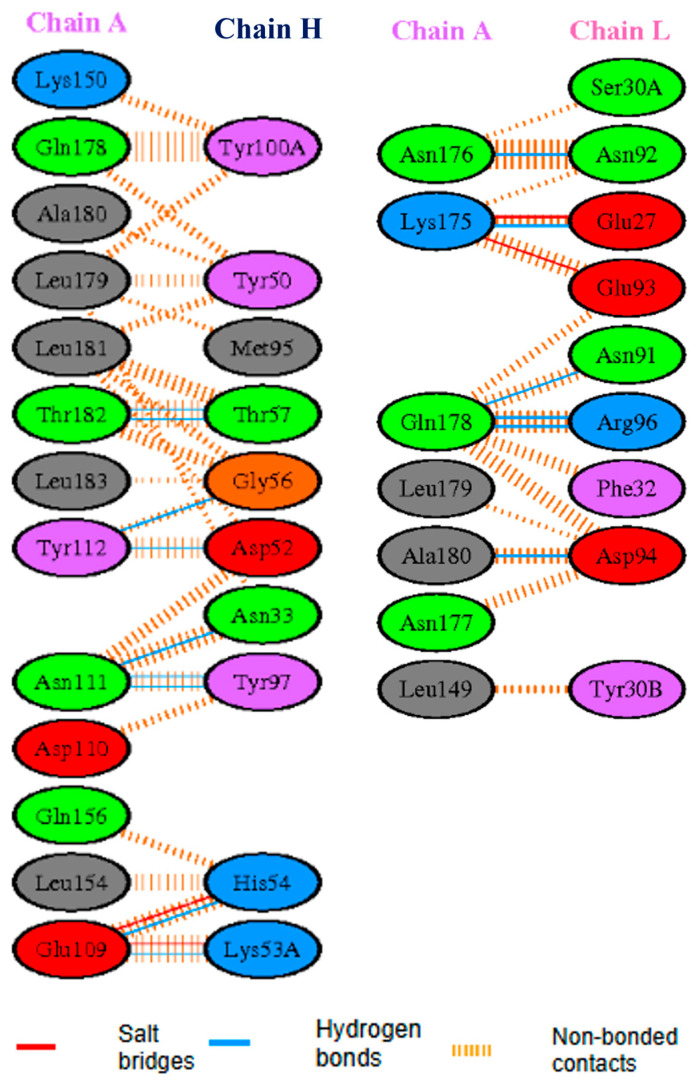
The detailed interaction map between the HER2 receptor and the anti-HER2 antibody H2Mab-119, sourced from PDBsum. The heavy chain (H chain) and light chain (L chain) of the antibody are shown in interaction with HER2, highlighting key residues that contribute to binding affinity and specificity.

Further, the PDBsum database was used to generate comprehensive interaction maps, emphasizing crucial residues involved in binding. As shown in Figure 1, the protein’s heavy chain (chain H), namely residues from Tyr50 to Thr57, which include the non-standard residue Lys53A, were found to have substantial interactions with HER2. The residues involved in the contact were Tyr50, Ile51, Asp52, Pro53, Lys53A, His54, Gly55, Gly56, and Thr57. There were a total of nine essential residues for this interaction. Similarly, the light chain (L chain) consisted of six crucial residues, namely Asn91, Asn92, Glu93, Asp94, Leu95, and Arg96. Earlier work in this domain demonstrated that these regions play a crucial role in the high-affinity binding seen in the antibody–HER2 complex. The heavy chain residues Lys53A and His54 formed H-bonds with Glu109 and the residues Asn33 and Tyr97 formed H-bonds with Asn111; moreover, Asp52 and Gly56 formed H-bonds with Tyr112, and lastly, Thr57 formed a H-bond with Thr182A. Further, the light-chain residues Asn92, Glu27, and Asp94 created H-bonds with Asn176, Lys175, and Ala180, respectively; similarly, Asn91 and Arg96 also formed H-bonds with Gln178. The PDBsum analysis showed a consistent pattern of preserved interactions, indicating potential binding sites for peptides.

### 3.2. Peptide Design

**Figure 2:**
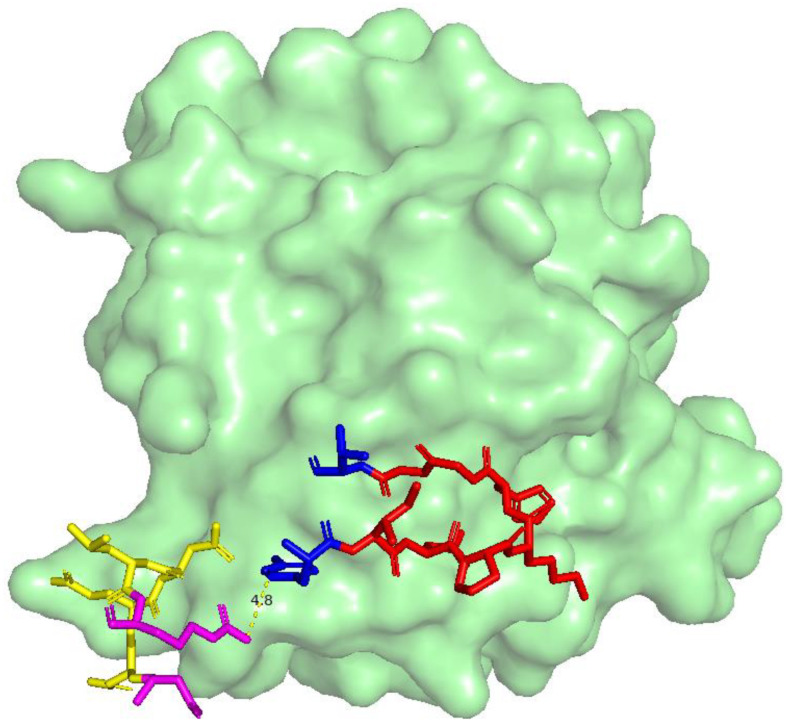
The HER2 receptor (green surface) and the anti-HER2 antibody H2Mab-119, highlighting key binding residues. Red segments represent heavy-chain residues, blue marks the terminal residues of the heavy chain, yellow indicates light-chain residues, and magenta marks the terminal residues of the light chain. The 4.8 Å distance between the nearest terminal residues of the heavy and light chains guided the design of a Gly–Ser (GS) linker, ensuring optimal spatial orientation in the final peptide sequence.

The amino acid sequences for the heavy chain (H chain) and light chain were specifically designed to optimize interactions with HER2. The heavy-chain sequence, ranging from residues Tyr50 to Thr57, was YIDPKHAGT, while the light-chain sequence, ranging from residues Asn91 to Arg96, was NNEDLR. These particular sequences were selected for their strong binding interactions with HER2, which is crucial for the effectiveness of the peptide. The heavy chain and the light chain were visualized using the PyMOL, as shown in Figure 2. The gap between the two chains was 4.8 Å. In protein structures, a gap of around 4.8 Å is within the range of distances that can be spanned by certain types of non-covalent interactions, such as hydrogen bonds or ionic interactions. These interactions typically occur between specific atoms on the side chains of two amino acids [28,29]. This distance is too short to accommodate more than two amino acid residues directly. Thus, the 4.8 Å gap indicates that only two amino acid residues are required because it represents a distance that can be effectively spanned by a single interaction between the side chains of those two residues. The interaction is likely strong and specific enough that no additional residues are necessary to stabilize or bridge this gap. Thus, to link the two sequences together, a Gly–Ser (GS) linker was employed. It was selected specifically for its remarkable flexibility and ability to span the 4.8 Å distance between the terminal residues, as shown in Figure 2. The flexibility of the GS linker is crucial because it allows the peptide to adjust and fit seamlessly into the binding site without imposing strain on the overall structure. This adaptability ensures that the peptide retains its proper conformation, which is essential for maintaining the structural integrity needed for effective binding interactions. By using the GS linker, the peptide was able to achieve an optimal alignment, enhancing its overall binding efficiency and functional performance. Consequently, the final peptide sequence, incorporating these optimized residues, was YIDPKHGGT-GS-NNEDLR.

### 3.3. Point Mutant Variants

In the course of the development of a comprehensive library of mutated peptide sequences, a point mutation approach was implemented using the original peptide sequence YIDPKHGGT-GS-NNEDLR. Here, each amino acid from the original peptide sequence was substituted with the 20 standard amino acids except for the original amino acid at that specific position. By applying this method to every position in the peptide, a set of 323 mutated sequences was generated, each representing a unique single-point mutation, as detailed in Supplementary Sheet S1. This comprehensive library encompasses all possible variations that could arise from substituting just one amino acid at a time, providing a broad spectrum of potential peptide sequences for further analysis and optimization.

### 3.4. Peptide Screening Using Deep-Purpose Machine Learning Model

This study used machine learning (ML) models to predict the binding affinities of an extensive library of variant peptides, primarily to accelerate the peptide screening process and identify high-affinity candidates. This study employed machine learning models trained on established binding affinities to efficiently and accurately evaluate novel peptide sequences, prioritizing options with the highest binding potential to the target protein. The ML technique facilitated data-driven insights into the correlations between the peptide sequence characteristics and binding affinity, providing a more profound comprehension of the determinants affecting robust interactions. The integration of speed, precision, and data insights offered by the ML models markedly improved the capacity to effectively investigate and assess peptide candidates for prospective therapeutic uses.

Here, the peptide screening method successfully assessed variants’ potential using sequence-encoding techniques and later predicted protein–protein interactions through the “Conjoint Triad” method and employed pre-trained models. Several machine learning models were then trained to predict the binding affinities of these peptides with a high accuracy. The performance of the models was evaluated using the R-squared, MSE, MAE, and correlation metrics, as shown in Table 1. The XGBoost model attained the highest training R^2^ score of 0.99, signifying an exceptional fit to the training data, closely succeeded by the ridge model at 0.95 and random forest model at 0.93. Gradient boosting had a somewhat reduced training R^2^ of 0.88. The XGBoost and random forest models exhibited comparable performances on the testing data, both achieving R^2^ scores of 0.67. The gradient boosting achieved a testing R^2^ of 0.64. The ridge regression, however, exhibited a substantial underperformance with a negative R^2^ score (−1.48), indicating potential difficulties in generalizing to fresh data.

Overall, the combination of high-quality data, careful feature selection, hyperparameter optimization, and the use of sophisticated models capable of capturing non-linear interactions likely contributed to the high R-squared values observed. The dataset used to train the models was carefully curated, ensuring diversity in peptide–protein binding interactions while maintaining a consistent and high-quality set of binding affinity values. The dataset’s high-quality and appropriate representation of binding variations might have contributed to the enhanced model performance. All models exhibited equal training and testing MSE values, suggesting uniform baseline error configurations. The elevated testing MSE across the models may suggest that none fully encapsulated all variations in the data; yet, they offered a satisfactory approximation. The mean absolute error (MAE) remained uniform across all models, indicating comparable accuracy in their absolute error rates. The same MAE suggests that although the ridge regression faced challenges with variation (as indicated by its R^2^) it did not exhibit markedly more absolute prediction errors. The training and testing correlations were same across all models, at 0.9733 for the training and 0.825 for the testing, signifying that each model exhibited a comparable level of linear relationship between the predicted and actual values. The XGBoost model demonstrated superior performance, attaining a highest training R^2^ of 0.99 and a competitive testing R^2^ of 0.67, while avoiding overfitting. Due to its greater performance, the XGBRegressor model was selected for further optimization. The XGBRegressor model was then used for the final screening of peptides, providing valuable insights into their binding affinities.

There were 323 different mutant peptide sequences, on which the optimized XGBRegressor model was used to predict the binding affinities for each sequence. The analysis indicated several peptides with substantial binding affinities, suggesting a high likelihood of efficient interaction with the target protein. Table 2 shows the top 20 peptides with the highest binding affinities that were selected for additional molecular docking based on the screened sequences. Also, the Supplementary Sheet S2 shows the 323 peptide sequences and their corresponding predicted values. The peptides with a strong affinity were the most promising options for improving protein stability by specifically attaching to them.

### 3.5. Peptide 3D Structure Modeling

The 20 peptide sequences identified during the screening process were modeled using the PEP-FOLD4 server, a robust tool specifically designed for generating accurate three-dimensional structures of peptides. PEP-FOLD4 predicts the most likely conformations of peptide sequences by combining a novel method that combines fragment assembly techniques with a coarse-grained force field. The server generated several structural models for each of the 20 peptides, providing a wide range of possible conformations. These models were then evaluated based on their stability and folding energy, two critical factors that influence a peptide’s structural integrity and functionality [30]. The most highly ranked models, those exhibiting the greatest stability and the lowest folding energy, were selected for further investigation through molecular docking studies. The selection of these top models was crucial because their stable conformations made them prime candidates for docking studies, where their ability to bind effectively to the target protein could be thoroughly assessed.

### 3.6. Docking and FreeSASA Study

**Figure 3:**
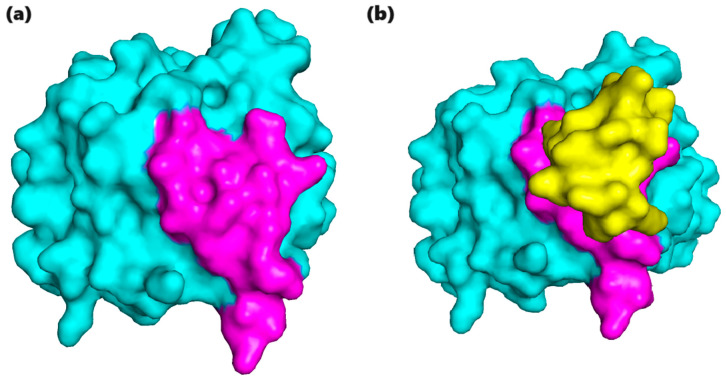
(**a**) A surface representation of the protein (cyan) highlighting the binding site (magenta) prior to docking. (**b**) A surface representation of the protein after docking, showing peptide 1 with best model (model_15) (yellow) bound to the protein, occupying and covering the binding site.

The 100 docking models for each of the 20 peptides, which were obtained from the HDOCK server, were examined for each peptide–protein complex. The use of FreeSASA was crucial in determining the precise measurement of the protein’s surface area that was buried upon peptide binding. This measurement acted as a reliable indicator of the robustness and durability of the interaction. The most suitable model for each peptide–protein complex could be discovered by calculating the delta SASA, which represented the difference in the surface accessibility between the bound and unbound states. The model with significant decrease in surface accessibility indicated the strongest and most stable binding association. The selected models, characterized by the largest delta SASA, were the most promising candidates. The best docking model from each of the 100 peptides is listed in Table 3, along with the bound and unbound SASA values and the change in the surface area when the peptide is bound. The delta SASA represents the difference between the bound and unbound SASA at the binding site, indicating the extent of surface area buried upon peptide binding. A higher delta SASA suggests a stronger and more stable interaction between the peptide and the target protein, making these models the most promising candidates for further analysis and therapeutic development. The bound and unbound state of the best model of peptide 1 is given in Figure 3. Figure 3a shows the binding site of the protein in magenta, illustrating the region that is available for potential interaction with a peptide. This represents the state of the protein before any peptide has bound, showing the unoccupied binding site in its natural form. In Figure 3b, the protein is shown after the docking process, where peptide 1 (depicted in yellow) has successfully bound to the protein. The peptide is now positioned over the binding site, effectively covering it. This demonstrates the interaction between the peptide and the protein, showing how the peptide occupies and interacts with the specific binding site.

### 3.7. Binding Free Energy

Energy minimization was important to optimize the structures and eliminate any steric clashes, guaranteeing that the complexes achieved a stable and realistic conformation. The calculated free binding energies provided crucial insights into the strength and stability of the peptide–protein interactions, with lower (more negative) binding energy values indicating stronger interactions.

Table 4 shows the free binding energies of various peptide–protein complexes, calculated using the MM-PBSA method after energy minimization. The results show a wide range of binding affinities, with negative values indicating stronger binding interactions. The peptide pep-17 exhibited the most favorable binding with a free energy of −9.27 kcal/mol, followed by pep-7 with −6.35 kcal/mol. In contrast, peptides such as pep-6 and pep-10 showed the least favorable interactions, with free energies of 45.79 and 25.64 kcal/mol, respectively. The data suggest a significant variation in binding strength across the peptides. Based on these results, the top four peptides, pep-17, pep-7, pep-2, and pep-15, with the most favorable binding energies were selected for further molecular dynamics (MD) analysis. These peptides exhibited the strongest and most stable interactions with the target protein.

### 3.8. Density Functional Theory (DFT)

The DFT study yielded information regarding the overall energy, energy levels of the highest occupied molecular orbital (HOMO), lowest unoccupied molecular orbital (LUMO), and amplitude of the dipole moment for each peptide. Understanding the electrical behavioral and potential reactivity of peptides upon docking needs a detailed analysis of these features.

The DFT calculations revealed significant variations in the electronic properties of the four peptides. Among the selected peptides, as shown in Table 5, pep-17 exhibited the most stable structure with the lowest DFT energy of −5788.50 kcal/mol. This stability was further supported by its moderate HOMO-LUMO gap, indicating a balanced reactivity profile. In contrast, pep-7 displayed a considerably higher dipole magnitude of 10,761.58 Debye, suggesting a strong polar character that could affect its interaction with surrounding molecules in a biological environment. The HOMO and LUMO energies also differed among the peptides, with pep-2 having the highest HOMO energy (0.6215 eV) and pep-15 having the lowest LUMO energy (0.0221 eV), which may have corresponded to differences in their chemical reactivities.

### 3.9. Molecular Dynamics Simulation (300 ns)

**Figure 4:**
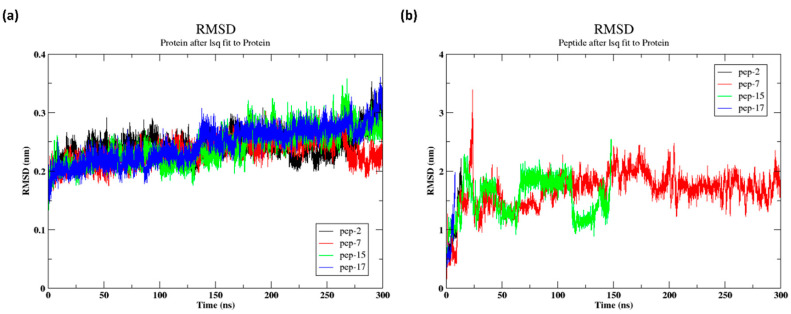
RMSD trajectories for the protein and peptides for the 300 ns simulation (**a**) The RMSD of the protein and (**b**) the RMSD of the peptides.

The root mean square deviation (RMSD) was calculated for the 300 ns simulation in order to evaluate the stability of their interaction with HER2. Figure 4a shows the RMSD of the protein when a peptide (pep-2, pep-7, pep-15, and pep-17) was bound. The protein showed an initial stabilization phase within the first 50 ns, with RMSD values fluctuating around 0.2 to 0.25 nm. Beyond this point, the RMSD gradually increased, especially after 200 ns, indicating potential structural changes or flexibility in the protein. The variation among the peptides suggests that each peptide may have influenced the protein’s stability and conformation differently over time. The RMSD of the protein increased to 0.3 nm when pep-17 and pep-2 were bound, while the RMSD of the protein when pep-15 was bound increased from 0.2 nm to 0.27 nm. The RMSD of the protein when pep-7 was bound remained constant at 0.2 nm, indicating minimal deviations.

Further, Figure 4b shows the RMSD of the peptides. The peptides occasionally appeared to move away from the binding pocket of the protein during the course of the simulation. The peptides started to move away from the binding pocket for pep-17 at around 9 ns, for pep-2 at around 14 ns, and for at pep-15 around 148 ns, while pep-7 remained within the binding pocket. Thus, those peptides (pep-2, pep-15, and pep-17) were not taken into consideration for the further analysis. Peptide 7 showed a higher deviation to 3.5 nm at 24 ns and then started to decrease to 1.5 nm. Further, it became stable around 1.7 nm at 200 ns.

**Figure 5:**
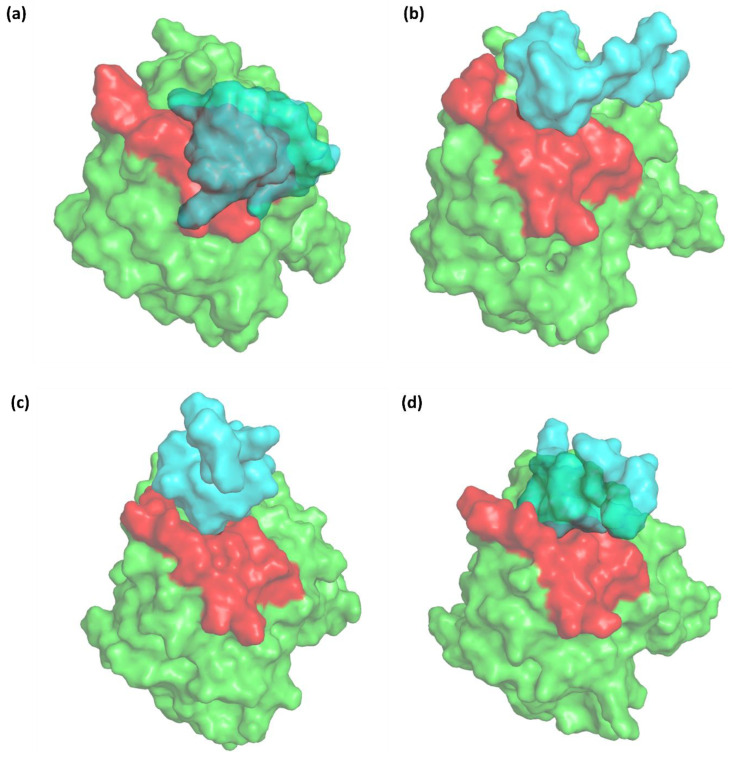
Conformational changes in the protein–peptide complex at different time points during molecular dynamics simulation: (**a**) 0 ns, (**b**) 100 ns, (**c**) 200 ns, and (**d**) 300 ns. The protein is shown in green, the binding site in red, and the peptide in cyan.

The trajectory of the selected protein–peptide complex was extracted at 0 ns, 100 ns, 200 ns, and 300 ns in the MD simulation. Figure 5 shows the progression of the protein–peptide binding interaction over 300 ns. At 0 ns, as seen in Figure 5a, the peptide was initially well positioned within the binding site. By 100 ns, as seen in Figure 5b, the peptide began to shift slightly but still maintained significant contact with the binding site. At 200 ns, as seen in Figure 5c, there was a more noticeable conformational adjustment in the protein, possibly indicating the increased flexibility or movement of the peptide within the binding pocket. Finally, at 300 ns, as seen in Figure 5d, the peptide appeared to be firmly nestled within the binding site, with significant surface contact, suggesting a stable interaction. This progression illustrates how the binding dynamics evolved over time, leading to a stable protein–peptide complex by the end of the simulation.

**Figure 6:**
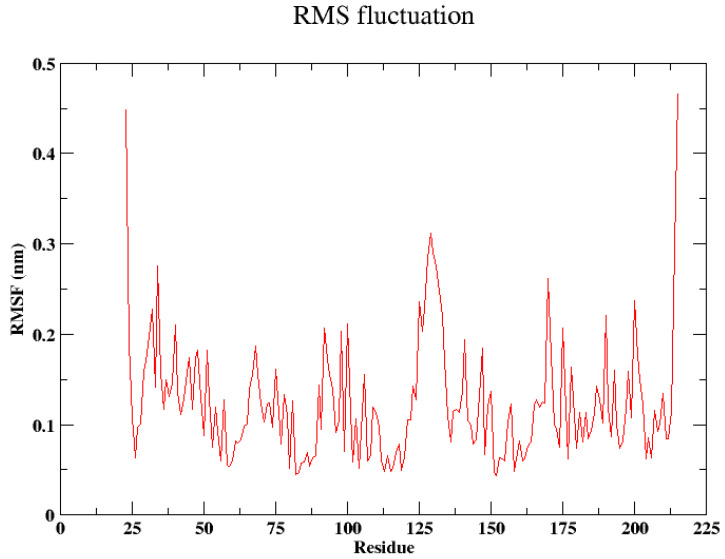
Root mean square fluctuation (RMSF) of protein when pep-7 was bound.

Later, the root mean square fluctuation (RMSF) was also analyzed, and it is given in Figure 6. The residues in the central region (approximately residues 25–200) generally showed lower RMSF values, typically below 0.3 nm, indicating more stability and less flexibility throughout the simulation. Peaks in this central region suggest localized areas of flexibility, potentially corresponding to loops or other mobile regions within the protein structure. The residues Val129 and Thr130 showed higher fluctuations compared to other residues, as indicated by the peaks in the RMSF plot. This suggests that these specific residues may have been part of a more flexible region in the protein, such as a loop or a hinge region, which allowed for greater movement. The increased flexibility of Val129 and Thr130 could have been critical for the protein’s function, possibly contributing to the dynamics of the active site, interaction with other molecules, or overall structural adaptability.

**Figure 7:**
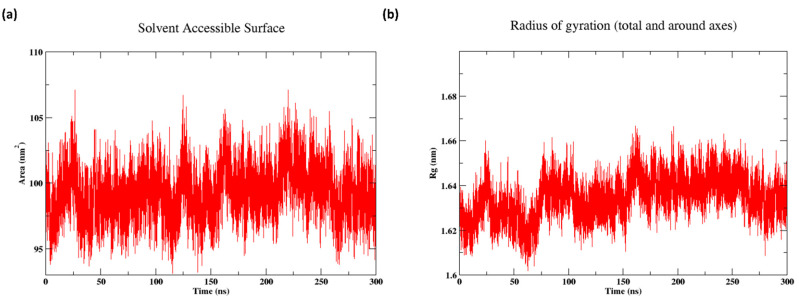
(**a**) Solvent-accessible surface (SASA). (**b**) Radius of gyration (Rg).

Figure 7a shows the solvent-accessible surface area (SASA) of the protein HER2, which reveals how the protein was exposed to the solvent while interacting with different compounds. Here, the SASA values fluctuate within a narrow range, generally between 95 and 105 nm^2^. The fluctuations indicate variations in the protein’s exposure to the solvent over time, reflecting the dynamic nature of the protein’s conformation during the simulation. The graph shows that while there are periods of relative stability around 100 nm^2^, there are frequent sharp spikes both above and below this value, suggesting continuous and significant conformational changes. These fluctuations were consistent throughout the simulation period, indicating ongoing interactions between the protein and the surrounding solvent.

Figure 7b shows the radius of gyration (Rg) of the system over 300 ns, indicating the compactness and overall size of the molecular structure throughout the simulation. The Rg values fluctuated between 1.61 and 1.66 nm. The consistent fluctuations suggest that the protein underwent minor conformational changes, affecting its overall compactness, but without significant expansion or contraction. These variations in the Rg reflect the dynamic nature of the molecular structure as it maintained a relatively stable size while adjusting slightly in response to internal and external interactions. This indicates that the protein did not significantly unfold or collapse during the simulation, maintaining its structural integrity.

**Figure 8:**
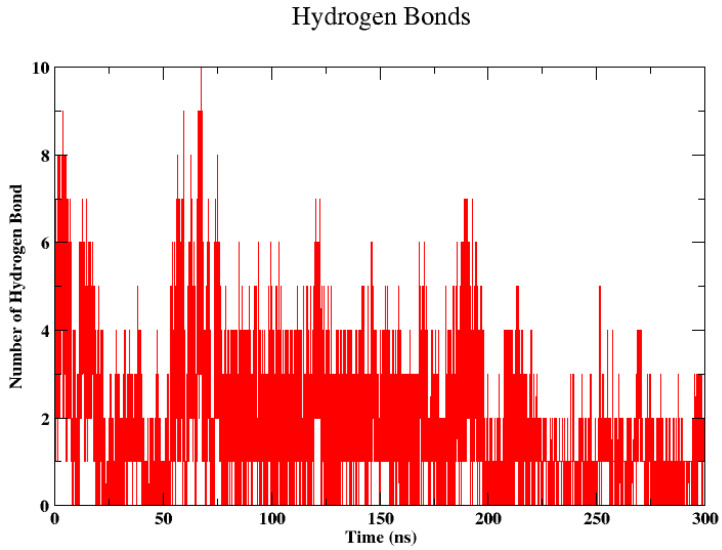
Hydrogen bond formed between HER2 and peptide 7 during 300 ns of simulation.

The formation of hydrogen bonds (H-bonds) between proteins and ligands plays a crucial role in stabilizing their interactions. These chemical bonds play a critical role in ensuring ligand recognition and complex stability by providing specificity and binding affinity and inducing conformational changes. Figure 8 shows fluctuations in the number of hydrogen bonds, which ranged from zero to ten, over a time span of 300 nanoseconds. Initially, the number of hydrogen bonds peaked at around eight then decreased and fluctuated between two and six for the majority of the timeline, with occasional spikes reaching up to eight bonds. The density of the bonds indicated more frequent changes in the number of hydrogen bonds in certain periods, particularly in the first 100 nanoseconds, after which the frequency of changes seemed to decrease, becoming more sporadic as time progressed.

**Figure 9:**
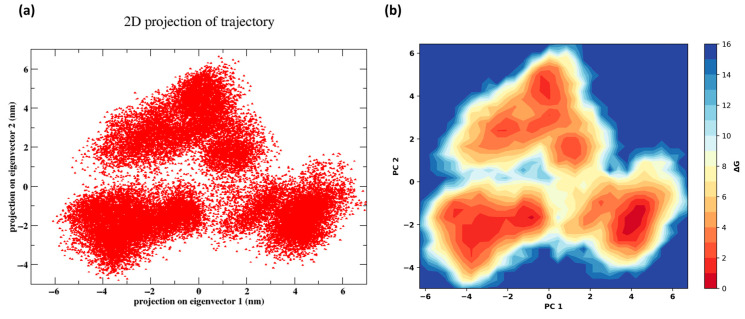
(**a**) Principal component analysis (PCA) of the protein–peptide complex. (**b**) Free energy landscape of the protein–peptide complex.

Figure 9a shows the principal component analysis (PCA). The plot reveals three distinct clusters of points, indicating that the system sampled three major conformational states during the simulation. The spread and density of the points within each cluster suggest varying degrees of flexibility or stability in these conformational states. Transitions between these states are visible as points connecting the clusters, indicating occasional shifts from one state to another. This type of PCA is useful for understanding the dominant motions in the system and identifying the key conformational changes that occur over the course of the simulation. Similarly, Figure 9b shows the free energy landscape (FEL). The color gradient, ranging from red (low free energy) to blue (high free energy), illustrates the relative stability of the different conformational states of the system. The red regions indicate the most stable conformational states, characterized by the lowest free energy, while the blue regions correspond to higher-energy, less stable states.

The FEL shows three major basins, each representing a distinct stable conformation that the system could occupy. These basins are separated by higher-energy barriers, indicating that transitions between these conformations require overcoming significant free energy barriers. The depth and spread of the basins reflect the stability and variability of each conformational state, with deeper basins being more stable and having lower energy. The visualization of these energy minima and the pathways connecting them provides valuable insights into the dynamic behavior and the conformational flexibility of the system under study.

**Figure 10:**
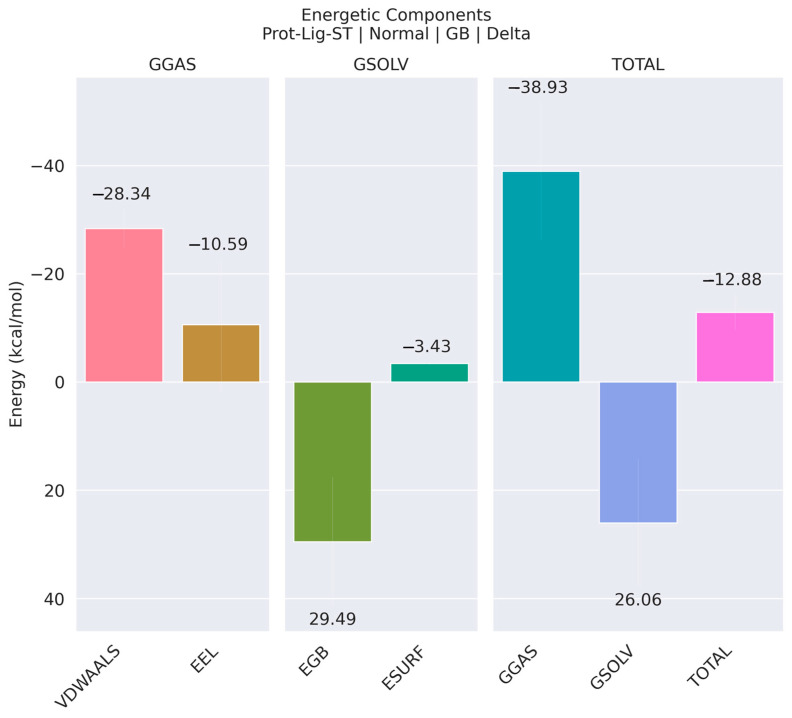
MM/GBSA binding free energies of complex in last 50 ns time-frame of total 300 ns in simulation.

In addition to the FEL, the MM/GBSA energy for the last 50 ns of the simulation was also computed for the complex, as shown in Figure 10. Figure 10 shows the energetic components contributing to the protein–ligand binding energy, divided into van der Waals (VDWAALS), electrostatic (EEL), polar solvation (EGB), and non-polar solvation (ESURF) energies, along with their total contributions. The van der Waals interactions (VDWAALS) and electrostatics (EEL) provided stabilizing contributions of −28.34 kcal/mol and −10.59 kcal/mol, respectively, under the GGAS category. In contrast, the polar solvation energy (EGB) under GSOLV contributed a destabilizing effect of 29.49 kcal/mol, partially offset by the non-polar solvation energy (ESURF) at −3.43 kcal/mol. The total energy for GGAS was −38.93 kcal/mol, while for GSOLV, it was 26.06 kcal/mol, resulting in a net total binding energy of −12.88 kcal/mol, indicating an overall favorable binding interaction despite the destabilizing solvation energy components. The optimized peptide, pep-7, demonstrated a net total binding energy of −12.88 kcal/mol, indicating a favorable binding interaction overall. In comparison, previous studies have reported the binding free energy of trastuzumab to be −12.0 kcal/mol [31], suggesting that pep-7 may exhibit better binding affinity than trastuzumab. Another study estimated the binding free energy (ΔG-bind) of trastuzumab to be −11.0 kcal/mol [32], while yet another reported a ΔG-bind value of −11.1 kcal/mol [33], further reinforcing the higher binding affinity of pep-7.

#### QM/MM

The QM/MM hybrid method is particularly useful for studying large systems, such as biomolecules or materials, where it is necessary to accurately describe a small, critical region (e.g., a chemical reaction site) while treating the rest of the system in a more computationally efficient manner [34]. The complex pep-7 is divided into two regions: a QM region, where electronic effects are critical (e.g., the active site or a reaction center), and an MM region, where classical force fields sufficiently describe the surrounding environment (e.g., solvent molecules or protein residues). The QM/MM interface handles the interactions between these regions, ensuring a smooth transition between quantum and classical descriptions.

**Figure 11:**
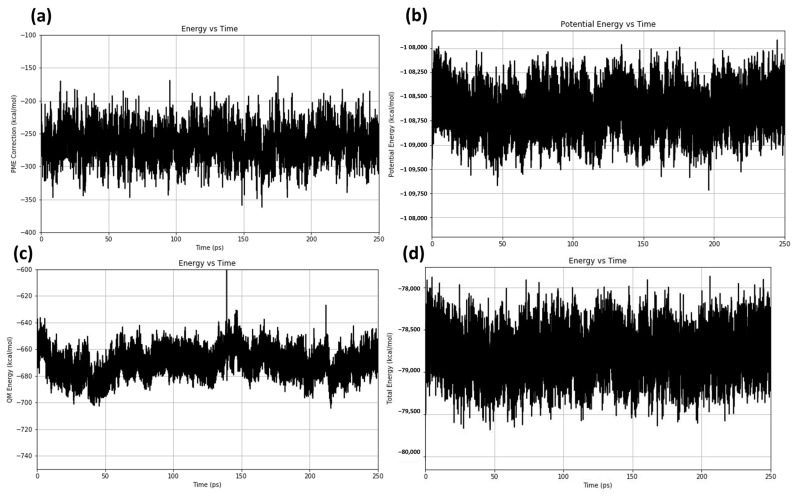
Energy analysis for protein–peptide complex in QM/MM simulation: (**a**) PME correction; (**b**) potential energy; (**c**) QM energy; (**d**) total energy.

The particle-mesh Ewald (PME) correction energy fluctuations across the simulation time are plotted in Figure 11a. The long-range electrostatic interactions computed using the PME approach exhibited fluctuation, as evidenced by the PME correction energy fluctuating between roughly −400 and −150 kcal/mol. This indicated dynamic changes in electrostatic interactions as the system equilibrated and is common in QM/MM simulations. The PME correction was stable and contributed to a constant electrostatic environment, as evidenced by the steady fluctuation with few deviations. Potential energy fluctuations during the simulation are plotted in Figure 11b, which shows that the system’s potential energy was stable. The potential energy fluctuated within a comparatively small range, staying constant at about −108,000 kcal/mol. This potential energy stability indicates that there were not any significant structural changes or destabilizing processes during the simulation, indicating that the protein–peptide complex was in a stable configuration. Figure 11c displays the quantum mechanics (QM) energy profile, which sheds light on the QM contributions to the protein–peptide interaction across time. Variations in the QM energy profile fell between −740 and −600 kcal/mol. The stable oscillation pattern indicates that the protein–peptide interaction’s quantum mechanical component stayed constant. This suggests that a stable association was supported by the fact that the electronic environment in the QM region was not changing significantly, which may include important components of the binding interface or catalytic residues. The protein–peptide complex’s total energy variations are plotted in Figure 11d, which illustrates the simulation’s overall energy stability. Overall stability in the protein–peptide complex was demonstrated by the total energy fluctuating around −79,000 kcal/mol. The overall energy consistency indicates that the QM and MM regions were interacting harmoniously, indicating that the parameters and QM/MM partitioning used successfully preserved a balanced simulation free from disruptive energy spikes. Overall, the protein–peptide combination was shown to be stable across the simulation time (250 ps) by the energy profiles across the PME correction, potential, QM, and total energy. This stability indicates that the complex was well-equilibrated, and the protein–peptide interactions were realistically represented by the QM/MM set up. The lack of significant energy fluctuations indicates that the complex did not undergo substantial conformational or electronic alterations, rendering this configuration appropriate for examining intricate interactions within the binding site and for subsequent drug design.

## 4. Discussion

Cancer remains a significant global health concern. The HER2 protein plays a critical role in regulating cell growth and proliferation, and its overexpression is associated with increased cell proliferation, invasiveness, and poor prognosis in cancer [35]. This study highlights the importance of HER2 as a crucial therapeutic target in various cancer types, including lung, breast, and gastric cancers. It outlines a thorough methodology for the design and optimization of peptide inhibitors through sophisticated computational approaches, pinpointing potential candidates with elevated binding affinities and stabilities. This study’s peptides target the HER2 receptor, which is overexpressed in various malignancies, highlighting their extensive application. HER2 mutations and overexpression, while predominantly linked to breast cancer, are also present in other solid tumors, including lung and stomach cancers, where they exacerbate tumor aggressiveness and lead to inferior outcomes [13,36,37,38]. Individuals with HER2-positive tumors, irrespective of malignancy classification, frequently encounter restricted targeted therapeutic alternatives. The creation of innovative therapeutics, such as the peptide inhibitors suggested in this study, may provide a viable alternative to current treatments like trastuzumab, which has shown inconsistent effectiveness outside breast cancer. The identified peptides, particularly pep-7, possess potential for wider application in HER2-overexpressing cancers. This study, unlike conventional peptide screening methods, integrates machine learning models to enhance prediction accuracy and efficiency [39]. Additionally, the use of advanced computational techniques such as MM/PBSA, DFT, and QM/MM simulations provided a comprehensive evaluation of peptide stability, binding energy, and electronic properties, surpassing standard docking and MD simulations [40,41]. The integration of machine learning (ML) with in silico analysis significantly enhances the drug discovery process. ML algorithms facilitate the analysis of large datasets to predict drug–target interactions, pharmacokinetics, and toxicity profiles. In silico techniques such as molecular docking, molecular dynamics simulations, and QSAR modeling enable the virtual screening of compound libraries, with ML refining predictive accuracy. The ML-driven optimization of molecular structures further aids in identifying lead compounds with improved efficacy and reduced adverse effects [42,43,44]. Additionally, this study specifically focuses on designing peptide inhibitors targeted at HER2-overexpressing lung cancer, filling a significant gap in existing HER2-targeted therapies, which are predominantly effective in breast cancer. This comparison highlights the novelty and potential efficacy of our integrated approach.

The study commenced with the extraction and examination of the HER2 protein structure, particularly emphasizing the crystal structure of HER2 complexed with the H2Mab-119 antibody. The interaction mapping of this complex highlighted essential residues in the heavy and light chains critical for binding, which formed the basis for the design of the peptide inhibitors. Utilizing PyMOL and PDBsum tools, the interaction sites were visualized, facilitating the systematic modification of the original peptide sequences to generate a library of variations [45,46]. XGBRegressor and other machine learning models were utilized to estimate peptide binding affinities, with the top 20 candidates being assessed by molecular docking and solvent accessibility analysis (FreeSASA) [47]. MM-PBSA calculations indicated that peptide 17 was the most potent HER2 binder, succeeded by peptides 7, 2, and 15, according to their negative free binding energies. Subsequent investigation utilizing DFT examined the electronic characteristics of these peptides, revealing that pep-17 exhibited the lowest energy and greatest stability, whilst pep-7 showed significant polarity with the highest dipole moment [48]. MD simulations spanning 300 ns demonstrated pep-7’s stable binding within the HER2 pocket, corroborated by the investigations of RMSD, RMSF, hydrogen bonding, and solvent accessibility. Principal component analysis and the free energy landscape elucidated stable structural states. The DFT calculations in this study were conducted without a solvent model, whereas the QM/MM simulations included a solvent environment. The QM/MM analysis emphasized the balance between molecular flexibility and electronic stability, offering valuable insights for designing peptide-based therapeutics and deepening the understanding of HER2-targeting peptide interactions. The QM/MM simulations in a solvent environment revealed that pep-7 exhibited stable binding characteristics, maintaining constant energy profiles across critical parameters including potential energy, QM energy, and total energy throughout the simulation period.

Although several HER2 inhibitors with established efficacy exist, this study employed a novel computational approach, integrating machine learning models such as DeepPurpose and QM/MM simulations to streamline and enhance the precision of peptide inhibitor design. Unlike conventional drug discovery methods that rely heavily on experimental screening, our approach allowed for the rapid identification of peptide candidates with strong binding affinities for the HER2 receptor. Overall, this study demonstrated that pep-7 is a promising candidate for stable interaction with the protein, providing a foundation for further exploration in therapeutic applications. Future experimental validation will further explore the therapeutic potential of these peptides in HER2-overexpressing cancers. This study not only advances HER2-targeting peptide design but also provides a foundation for future cancer therapeutics.

## 5. Method

### 5.1. Protein Structure Retrieval and Interaction Map

The information regarding the receptor tyrosine–protein kinase erbB-2 (HER2) was searched in UniProt [49], and the reviewed entry with UniProt ID P04626 was selected. The crystal structure with the best resolution was selected with PDB ID: 8JYR [50]. The crystal structure contained an antibody, H2Mab-119 (an inhibitor of HER2), in a complex with HER2. This structure was chosen due to the clarity of the HER2–antibody interface, which could serve as a reliable reference for designing peptide inhibitors. Further, the structure was visualized in PyMOL [51], and the PDBsum database was used to generate comprehensive interaction maps, emphasizing crucial residues involved in binding [45]. PyMOL enabled the detailed examination of the HER2–antibody binding interface, allowing for the clear identification of interaction regions. PDBsum provided a breakdown of protein–ligand and protein–protein interactions, highlighting hydrogen bonds, hydrophobic contacts, and other non-covalent interactions. This interaction map was used to pinpoint the specific amino acid residues in HER2 that interact directly with the antibody H2Mab-119. Further, the amino acid stretch, which was continuous and showed binding with HER2, was selected for the peptide designing; considering the PDB complex as a reference, this was treated as an original inhibitory peptide sequence. Utilizing this peptide sequence as a framework, additional alterations and optimizations could be implemented to improve binding affinity, stability, or other therapeutic attributes. This comprehensive methodology, encompassing structural selection, visualization, interaction mapping, and sequence selection, established a solid framework for the design of peptide inhibitors aimed at HER2, utilizing knowledge derived from a recognized HER2-inhibiting antibody.

### 5.2. Point Mutation for Peptide Generation

The initial peptide sequence was methodically altered by point mutations to create an extensive library of alternative peptide sequences. Every amino acid position in the original sequence was systematically replaced with each of the other 19 amino acids from the standard set of 20, excluding the original amino acid at that particular location. This method guaranteed a comprehensive examination of each site for alternative residues, resulting in a diversified collection of peptide variations. The library was further assessed for possible enhancements in binding affinity, stability, and other favorable attributes, facilitating the identification of optimized peptide candidates with improved therapeutic potential.

### 5.3. Machine Learning Using Deep Purpose

For the peptide screening method, the sequence-encoding technique was used to evaluate the potential of different variants of peptides. This encoding approach captures the physicochemical properties of adjacent amino acids, thereby preserving relevant sequence information for binding affinity prediction. The peptide sequences were encoded using the Conjoint Triad approach, which records amino acid characteristics to aid in interaction prediction in order to screen and forecast the binding affinities of peptide variants. The “Conjoint triad” [52] method was used to encode the target sequences to facilitate protein–protein interaction predictions. Robust evaluation was ensured by splitting the dataset 80:20 between training and testing. Four machine learning models, RandomForestRegressor, Ridge, GradientBoostingRegressor, and XGBRegressor, were used. Each model was fine-tuned with parameters (such as alpha = 1.0 for Ridge and 100 estimators and random state 42 for ensemble models) to maximize the performance, as shown in Table 6. The models’ prediction accuracy was evaluated using the R-squared metric after they were trained using encoded data. The top 20 peptides were subsequently analyzed based on their expected binding affinities, which facilitated the identification of potential HER2-targeting peptides. The efficacy of each model was evaluated using the R-squared, MSE, MAE, and correlation metrics, which quantifies the extent to which each model accounted for the variance in binding affinities among the peptide variants. This integration of encoding and machine learning models facilitated the rapid and precise selection of peptide candidates with significant binding potential for HER2.

### 5.4. Peptide Modeling

The PEP-FOLD4 [53] online server, which can be accessed at https://bioserv.rpbs.univ-paris-diderot.fr/services/PEP-FOLD4/ Accessed on 23 August 2024, was used to model the 3D structures of the top 20 peptide sequences identified during the screening procedure. This server generated three-dimensional structures for each peptide by combining fragment assembly with a coarse-grained force field. PEP-FOLD4 produced a series of probable structural models for each peptide sequence, offering multiple conformations for every peptide. This collection of structures illustrates the inherent flexibility and potential conformational states a peptide may assume in a biological context. Each generated model was evaluated using an internal scoring system, where lower scores generally signified more advantageous conformations in accordance with the applied force field.

### 5.5. Docking and FreeSASA Calculation

To conduct docking, the top 20 peptide models produced by PEP-FOLD4 were docked to the HER2 protein using the HDOCK server [54], which may be accessed at http://hdock.phys.hust.edu.cn/, accessed on 23 August 2024. The top docking models were obtained and examined for each peptide–protein complex. The HDOCK server is a comprehensively integrated set of tools for performing protein–peptide docking rapidly with high accuracy. This server uses a hybrid method of template-based and template-free docking approaches to predict receptor and ligand interactions based on the input 3D structure of receptor and ligand molecules. The water molecules were removed from the crystal structure, and other heteroatoms were also removed to clean the protein structure. The docking parameters were configured, and the number of models generated at the end of docking was set to 100. The docking process was initiated, and the calculations were performed for generating the top 100 docked poses.

The change in SASA (solvent-accessible surface area) can be used to find the binding interface of the antigen–antibody complex. Here, the SASA was calculated for each residue of the antigen–antibody docked complexes in the bound and unbound state using the FreeSASA tool [55] which is accessible at https://freesasa.github.io/, Accessed on 23 August 2024. This tool was specifically built for determining the surface area of large molecules. FreeSASA was used to compute the SASA for the residues in the binding site, both in the absence of any ligands (protein alone) and in the presence of a ligand (protein–peptide complex). FreeSASA uses a combination of Lee and Richards’ (L&R) algorithm [56] as well as Shrake and Rupley’s (S&R) algorithm [57]. Lee and Richards’ method involves approximating the molecular surface using a series of slices, while Shrake and Rupley’s approach approximates the surface of each sphere using a set of test points.

### 5.6. Binding Free Energy Calculation

The free binding energy was calculated to evaluate the intensity of the interaction between the peptides and the protein. The energy minimization of the protein–peptide complex was conducted using the GROMACS 2022.4 package [58]. CHARMM36 [59] was employed to establish the molecular topology and force field parameters, which were assigned to both the protein and peptides. Later, the generation of the force-field parameters and topologies of the compounds and the control inhibitor, the CGenFF [60] server, was used. The Ewald particle-mesh method was utilized in order to calculate the electrostatic force [61]. The system was solvated in the cubic box with the TIP3P water solvent model [62]. Na^+^ and Cl^−^ ions were subsequently introduced to perform the neutralization. Further, to eliminate steric conflicts, the system underwent 50,000 minimization steps utilizing the steepest descent (SD) method. Subsequently, the LINCS algorithm [63] was implemented to restrict the bonds, thus attaining system stability. Further, the MM/GBSA (molecular mechanics generalized Born surface area) technique was used in this study [64,65]. The GROMACS tool gmx_MM/PBSA was used to calculate the binding free energy of the complexes until the energy minimization. The equation involved in calculating binding free energy is described below:

(1) $$∆G=G_{complex}-[G_{receptor}+G_{ligand}]$$

(2) $${\Delta G}_{binding}=\Delta H-T\Delta S$$

(3) $$\Delta H=\Delta G_{GAS}+\Delta G_{SOLV}$$

(4) $$\Delta G_{GAS}=\Delta E_{EL}+\Delta E_{VDWAALS}$$

(5) $$\Delta G_{SOLV}=\Delta E_{GB}+\Delta E_{SURF}$$

(6) $$\Delta E_{SURF}=\gamma .SASA$$

In Equation (1), the variables *G_complex_*, *G_receptor_*, and *G_ligand_* represent the total free energies of the protein–peptide complex, the free enzyme, and the peptide in the solvent, respectively. The remaining equations in Equations (2)–(6) illustrate the alterations in the gas-phase energy, ΔG_GAS_, the solvation free energy change, ΔG_SOLV_, the conformational entropy change, –TΔS, and the enthalpy change, ΔH. The investigation incorporated the solvent-accessible surface area (SASA) and the solvent’s surface tension (γ), both of which were displayed graphically. Changes in electrostatic and van der Waals energies were denoted by ΔE_VDWAALS_ and ΔE_EL_, respectively. Furthermore, as indicated in the research, the polar and nonpolar solvation energy changes were accurately represented by ΔE_GB_ and ΔE_SURF_. The model which had the highest free binding energy was selected for molecular dynamics simulation.

### 5.7. DFT Calculation

One of the most used techniques for the ab initio computations of atoms, molecules, crystals, surfaces, and interactions is density functional theory (DFT) [66]. The most popular theoretical approach in quantum chemistry is DFT [67]. A Python program was used to calculate the DFT of each of the top peptides. For the electronic structure studies, a Python-based simulation of chemistry framework (PySCF) was used [68]. A density functional theory (DFT) analysis of the entire geometry was conducted us-ing the Becke 3-parameter Lee-Yang-Parr (B3LYP) functional, a hybrid functional that in-corporates a combination of exact Hartree–Fock exchange and DFT exchange–correlation terms [69], utilising a fundamental basis set of 6–31 G* [70].

### 5.8. Molecular Dynamics Simulation

The best model selected was used for the molecular dynamics simulation for 300 ns. The method, until energy minimization, is given in Section 3.3. Later, the temperature of the entire system was increased to 310 K with a timestep of 2 fs throughout a 100 ps simulation in the NVT ensemble. The coordinates of the structure were stored at 10 ps intervals for the duration of the production run, which spanned 300 ns. In addition, the velocity scaling [71] method was employed as a temperature coupling in order to maintain stimulation at a consistent temperature. The Parrinello–Rahman [72] pressure coupling method was subsequently implemented to maintain a constant pressure during the manufacturing process. Further post-MD analysis was carried out. The post-MD analysis was performed on the visual platform called “Analogue”, developed by Growdea Technologies [73,74] (https://growdeatech.com/Analogue/, accessed on 23 August 2024). The final conformation of the best compound was extracted for the QM/MM hybrid approach.

### 5.9. Quantam Mechanisc/Molecular Mechanics (QM/MM)

The QM/MM (quantum mechanics/molecular mechanics) approach is a computational technique used to study complex molecular systems by combining the accuracy of quantum mechanics (QM) with the efficiency of molecular mechanics (MM) [75,76]. The final conformation obtained from the classical MD simulation was first cleaned by removing water and ions. A small classical MD run was then set up using the QuickMD plugin of VMD [77]. The procedure began with loading the cleaned PDB file of the complex, which was thoroughly checked for any topological and parameter issues. The simulation was performed in an explicit solvent environment, using a TIP3P water model [78] within a 12 Å simulation box. NaCl was added at a concentration of 0.15 mol/L to ensure proper ionization. The simulation sequence included several stages: A minimization run of 2000 steps was conducted with restraints on the backbone in an NPT ensemble at 0 °C and a pressure of 1 atm. This was followed by an annealing run of 14,400 steps, also with backbone restraints, at 27 °C and a pressure 1 atm. Finally, an equilibration run of 50,000 steps was performed under the same conditions. All these simulations were executed using the NAMD software version 3.0.

After the equilibration run, a QM/MM simulation was set up. The procedure began by loading the QuickMD file from the previous classical MD run, specifically using the last structure from the equilibration run. Molecular Orbital PACkage (MOPAC) [79] version 22.1.1 was employed as the QM software, and the QM region was defined by selecting the residue of the peptide that was forming an H-bond with the receptor, along with each atom within a 5-angstrom radius of this residue. The QM/MM simulation sequence included several stages. First, a QM/MM minimization run of 100 steps was conducted with restraints on the backbone in an NPT ensemble at 0 °C and a pressure of 1 atm. This was followed by a QM/MM annealing run of 720 steps under the same conditions but at 27 °C. A QMMM equilibration run of 100 steps was then performed, also at 27 °C and a pressure of 1 atm with backbone restraints. Finally, the main QM/MM run was conducted for 500,000 steps in an NPT ensemble at 27 °C and a pressure of 1 atm, with no atoms restrained during this phase. Further, the HOMO (highest occupied molecular orbital) and LUMO (lowest unoccupied molecular orbital) energy levels, dipole moments, and energy differences were calculated.

## 6. Conclusions

This study presents a comprehensive approach to designing and optimizing peptide inhibitors targeting the HER2 protein, a critical player in cancers such as breast and lung cancer. The study used a combination of molecular dynamics (MD) simulations or classical MD, density functional theory (DFT) calculations, a quantum mechanics/molecular mechanics (QM/MM) hybrid approach, and machine learning models to identify and analyze promising peptide candidates. The results highlighted the significance of key interactions between HER2 and the designed peptides, particularly focusing on the peptide pep-7, which demonstrated stable binding and favorable electronic properties. The study’s findings suggest that pep-7 could serve as a potential therapeutic agent, providing a solid foundation for further exploration in drug development. This study has enhanced the understanding of peptide–protein interactions through the integration of modern computational approaches, including QM/MM simulations. These findings provide a viable avenue for the development of targeted treatments for HER2-overexpressing cancers with potential applications in the treatment of both breast and lung cancer.

## Figures and Tables

**Table 1:** R-squared, MSE, MAE, and correlation scores of machine learning models for predicting peptide-binding affinities.

Scores	Random Forest	Ridge	Gradient Boosting	XGBoost
Training R2	0.9339	0.952	0.8868	0.9954
Testing R2	0.6723	−1.482	0.647	0.6733
Training MSE	33.2007	33.2007	33.2007	33.2007
Testing MSE	123.4048	123.4048	123.4048	123.4048
Training MAE	3.5479	3.5479	3.5479	3.5479
Testing MAE	7.1449	7.1449	7.1449	7.1449
Training Correlation	0.9733	0.9733	0.9733	0.9733
Testing Correlation	0.825	0.825	0.825	0.825

**Table 2:** The top 20 peptide sequences with the highest predicted binding affinities, as determined by the optimized XGBRegressor model.

Peptide	Sequence	Predicted Value
pep-1	YIDPKHGGTCSNNEDLR	−10.2406
pep-2	YIDPKHGGTDSNNEDLR	−10.2195
pep-3	YIDPKHGGTESNNEDLR	−10.2195
pep-4	YRDPKHGGTGSNNEDLR	−10.1626
pep-5	YKDPKHGGTGSNNEDLR	−10.1626
pep-6	YIDPKHGGTMSNNEDLR	−10.1174
pep-7	YIDPKHGGTSSNNEDLR	−10.1174
pep-8	YIDPKHGGTTSNNEDLR	−10.1174
pep-9	YIDPKHGGTYSNNEDLR	−10.1174
pep-10	YIDPKHGGTGRNNEDLR	−10.1014
pep-11	YIDPKHGGTGNNNEDLR	−10.1014
pep-12	YIDPKHGGTGQNNEDLR	−10.1014
pep-13	YIDPKHGGTGHNNEDLR	−10.1014
pep-14	YIDPKHGGTGKNNEDLR	−10.1014
pep-15	YIDPKHGGTGWNNEDLR	−10.1014
pep-16	YIDPKHGGTGDNNEDLR	−10.08
pep-17	YIDPKHGGTGENNEDLR	−10.08
pep-18	YIDPKHGGAGSNNEDLR	−10.0285
pep-19	YIDPKHGGGGSNNEDLR	−10.0285
pep-20	YIDPKHGGVGSNNEDLR	−10.0285

**Table 3:** The best docking models selected for each peptide out of 100 docked models based on the highest ∆SASA values, calculated using the FreeSASA program.

Peptide	Best Model	Unbound SASA (Å^2^)	Bound SASA (Å^2^)	∆SASA (Å^2^)
pep-1	model_15	1152.33	744.30	408.03
pep-2	model_22	1152.33	799.17	353.16
pep-3	model_60	1152.33	772.72	379.61
pep-4	model_32	1152.33	733.67	418.65
pep-5	model_1	1152.33	751.34	400.98
pep-6	model_10	1152.33	691.66	460.67
pep-7	model_6	1152.33	725.49	426.84
pep-8	model_25	1152.33	727.16	425.17
pep-9	model_43	1152.33	711.58	440.75
pep-10	model_34	1152.33	735.50	416.82
pep-11	model_3	1152.33	708.99	443.34
pep-12	model_22	1152.33	736.32	416.01
pep-13	model_9	1152.33	693.15	459.18
pep-14	model_2	1152.33	719.97	432.35
pep-15	model_7	1152.33	747.69	404.64
pep-16	model_11	1152.33	789.21	363.12
pep-17	model_26	1152.33	749.36	402.97
pep-18	model_4	1152.33	753.00	399.32
pep-19	model_15	1152.33	763.70	388.62
pep-20	model_38	1152.33	754.82	397.51

**Table 4:** The free binding energies (in kcal/mol) calculated for each peptide–protein complex using the MM-PBSA method after energy minimization. The top four peptide has been highlighted in the bold.

Peptide	Binding Free Energy
**pep-17**	**−9.27**
**pep-7**	**−6.35**
**pep-2**	**−1.52**
**pep-15**	**−1.41**
pep-3	0.2
pep-13	0.97
pep-12	3.42
pep-9	3.51
pep-14	4.56
pep-4	4.85
pep-8	4.97
pep-11	6.87
pep-19	7.94
pep-18	7.98
pep-16	8.16
pep-1	14.36
pep-5	15.25
pep-20	19.3
pep-10	25.64
pep-6	45.79

**Table 5:** DFT energy, HOMO-LUMO energies, and dipole magnitudes for selected peptides.

Peptides	DFT Energy	HOMO Energy	LUMO Energy	Dipole
pep-2	−5429.83	0.621	0.112	4563.60
pep-7	−3386.93	0.085	0.073	10,761.58
pep-15	−5040.84	0.018	0.022	5664.78
pep-17	−5788.49	0.508	0.054	2654.25

**Table 6:** Parameters for the four models used for optimization.

Machine Learning Model	Parameter	Value
RandomForestRegressor	Number of estimators	100
	Random state	42
Ridge	Regularization parameter (alpha)	1
GradientBoostingRegressor	Number of estimators	100
	Random state	42
XGBRegressor	Number of estimators	100
	Random state	42

## Data Availability

Data are contained within the article.

## Supplementary Materials

The following supporting information can be downloaded at: https://www.mdpi.com/article/10.3390/cancers16233979/s1, Supplementary Sheet S1: Detail of the 323 mutated sequences was generated, each representing a unique single-point mutation, Supplementary Sheet S2: Binding affinities of the 323 mutated peptides when docked with HER2.
